# Beyond descriptive SWOT in pharmacy strategy: A quantitative framework for leaders, managers and educators from prioritization to strategic response

**DOI:** 10.1016/j.rcsop.2026.100836

**Published:** 2026-08-24

**Authors:** Mohanad Odeh, Khaled A. Harb

**Affiliations:** a Department of clinical pharmacy and pharmacy practice, The Pharmaceutical science school, Zarqa, The Hashemite University, Jordan; b Dar Aldawa Development and Investment Co. LTD, Amman, Jordan

**Keywords:** SWOT analysis, Quantitative SWOT, Strategic planning, Pharmacy management, Pharmacy administration, Stakeholder analysis, Decision support

## Abstract

Strengths, Weaknesses, Opportunities, and Threats (SWOT) analysis is widely used in pharmacy strategy because it is simple, familiar, and adaptable across practice, education, and management settings. However, descriptive SWOT often stops at factor identification, offering limited support for prioritization, stakeholder comparison, and strategic action in multi-stakeholder pharmacy contexts.

This article develops a conceptual-methodological framework for advancing SWOT through quantification, stakeholder-sensitive comparison and strategic translation. An illustrative mixed-methods university–industry pharmacy education application is used only to demonstrate operationalization of the framework, serving as a methodological example rather than a definitive evaluation of a local program.

The framework advances descriptive SWOT through five stages. First, SWOT defines the focal strategic profile. Second, broader macro-context is distinguished from organization- or program-specific diagnosis through Political, Economic, Social, Technological, Environmental, and Legal (PESTEL) analysis. Third, factor importance is quantified using analysis-appropriate methods, ranging from ordinal rating and continuous scoring to weighting, sensitivity analysis, or uncertainty-sensitive approaches. Fourth, gap analysis identifies alignment or separation in how groups prioritize the same factors. Fifth, strategic response is developed through Threats, Opportunities, Weaknesses, and Strengths (TOWS) matrix translation, converting selected quantified findings into growth, reinforcement, defensive, and risk-reduction strategic options.

Overall, the framework advances SWOT from strategic diagnosis to measurable strategic response by integrating qualitative contextual mapping with structured measurement and analysis within a transparent, comparative, and decision-relevant pathway. It offers pharmacy leaders, managers, and educators a scalable approach for moving from descriptive SWOT toward prioritized and action-oriented strategic planning.

## 1 Introduction

Strategic planning has become increasingly important across contemporary pharmacy systems, including hospital pharmacy departments, community pharmacy services, pharmacy education, pharmaceutical distribution, and medicines policy implementation.^1^, ^2^, ^3^, ^4^ Pharmacy practice now operates within environments shaped by workforce constraints, service expansion, patient centralization, digital transformation, regulatory complexity, and rising expectations for measurable impact on healthcare outcomes, organizational performance, and health-system efficiency.^1^, ^2^, ^5^, ^6^

Strategic planning draws on a broad repertoire of tools, each serving a distinct strategic function. Competitive-positioning tools help organizations understand industry structure, advantage, and market space. These include Porter's Five Forces for analyzing competitive pressures within an industry,^7^ resource-based and Value, Rarity, Inimitability, and Organization (VRIO) approaches for identifying sources of sustained competitive advantage,^8^ Blue Ocean Strategy for pursuing value innovation and uncontested market space,^9^ and portfolio tools, such as the growth-share matrix, for guiding resource-allocation decisions across products, services, or business units.^10^ Market-growth and product, value-design tools address different strategic questions: the Ansoff matrix supports decisions about market penetration, market development, product development, and diversification,^11^ whereas the value proposition canvas helps clarify customer fit and value creation.^12^ Execution and performance-management tools, including the balanced scorecard, translate strategic intentions into measurable objectives and indicators,^13^ while objectives and key results support goal alignment and measurable execution.^14^ Scenario-planning approaches further help organizations examine uncertainty, anticipate future risks, and test strategic assumptions.^15^ Within the diagnostic family of strategic tools, Political, Economic, Social, Technological, Environmental, and Legal (PESTEL) analysis is used to examine macro-environmental conditions, whereas Strengths, Weaknesses, Opportunities, and Threats (SWOT) analysis provides a focal diagnostic structure for classifying organization- or program-specific factors as internal strengths and weaknesses or external opportunities and threats.^16^, ^17^ The present paper focuses on SWOT because it is one of the most widely used and accessible diagnostic tools in pharmacy-related planning, yet its common descriptive use can be strengthened through quantification, stakeholder comparison, and strategic translation.

In its conventional descriptive use, SWOT provides a useful structure for classifying strategic factors into two internal categories, strengths and weaknesses, and two external categories, opportunities and threats. However, descriptive SWOT does not inherently establish the relative importance and impact of these factors, reveal whether stakeholder groups interpret or prioritize them differently, or specify how the resulting profile should be translated into strategic action. This limitation should not be viewed as a failure of SWOT itself, but as a consequence of its deliberate simplicity and its frequent use as an unweighted, non-comparative listing exercise rather than as a measurable and structured decision-support process.^17^ The issue is particularly relevant in pharmacy contexts, where strategic decisions often depend on alignment among stakeholders with different responsibilities, priorities, and decision criteria.^3^, ^6^, ^18^, ^19^ Accordingly, strengthening SWOT for pharmacy strategy requires moving beyond factor identification toward clearer prioritization, more transparent stakeholder comparison, and a stronger link between diagnosis and action.^17^, ^20^, ^21^ This creates a methodological gap between descriptive strategic diagnosis and decision-oriented strategic planning, particularly in multi-stakeholder pharmacy settings.

Quantitative SWOT addresses this gap by adding structured ranking, scoring, weighting, or stakeholder-comparison procedures to the descriptive SWOT profile, thereby extending SWOT from a diagnostic framework into a more decision-oriented strategic planning approach. Prioritized SWOT findings can then be carried forward into a TOWS matrix, which matches strengths, weaknesses, opportunities, and threats to generate strengths–opportunities (SO), weaknesses–opportunities (WO), strengths–threats (ST), and weaknesses–threats (WT) strategy options. This sequence clarifies how quantitative SWOT can move from factor identification to prioritization, stakeholder comparison, and strategic response.

**The aim of this paper is twofold**. First, it develops a conceptual-methodological account of quantitative SWOT for pharmacy strategy, clarifying how SWOT can progress from descriptive factor identification toward structured prioritization, stakeholder comparison, and TOWS-based strategic translation. Second, it illustrates this progression through an embedded mixed-methods, qualitative–quantitative, cross-sectional stakeholder application involving university–industry collaboration in advanced pharmaceutical sales and marketing training in Jordan. This application is included to demonstrate how the method can be operationalized, not to position the manuscript as a case-study evaluation. The manuscript's contribution lies in connecting tools that are often discussed separately—PESTEL, descriptive SWOT, quantitative SWOT, stakeholder-gap analysis, and TOWS matrix—into a coherent framework for measurable, stakeholder-sensitive pharmacy strategic planning.

## 2 Conceptual roadmap for advancing descriptive SWOT toward quantitative strategic response

**Fig. 1:**
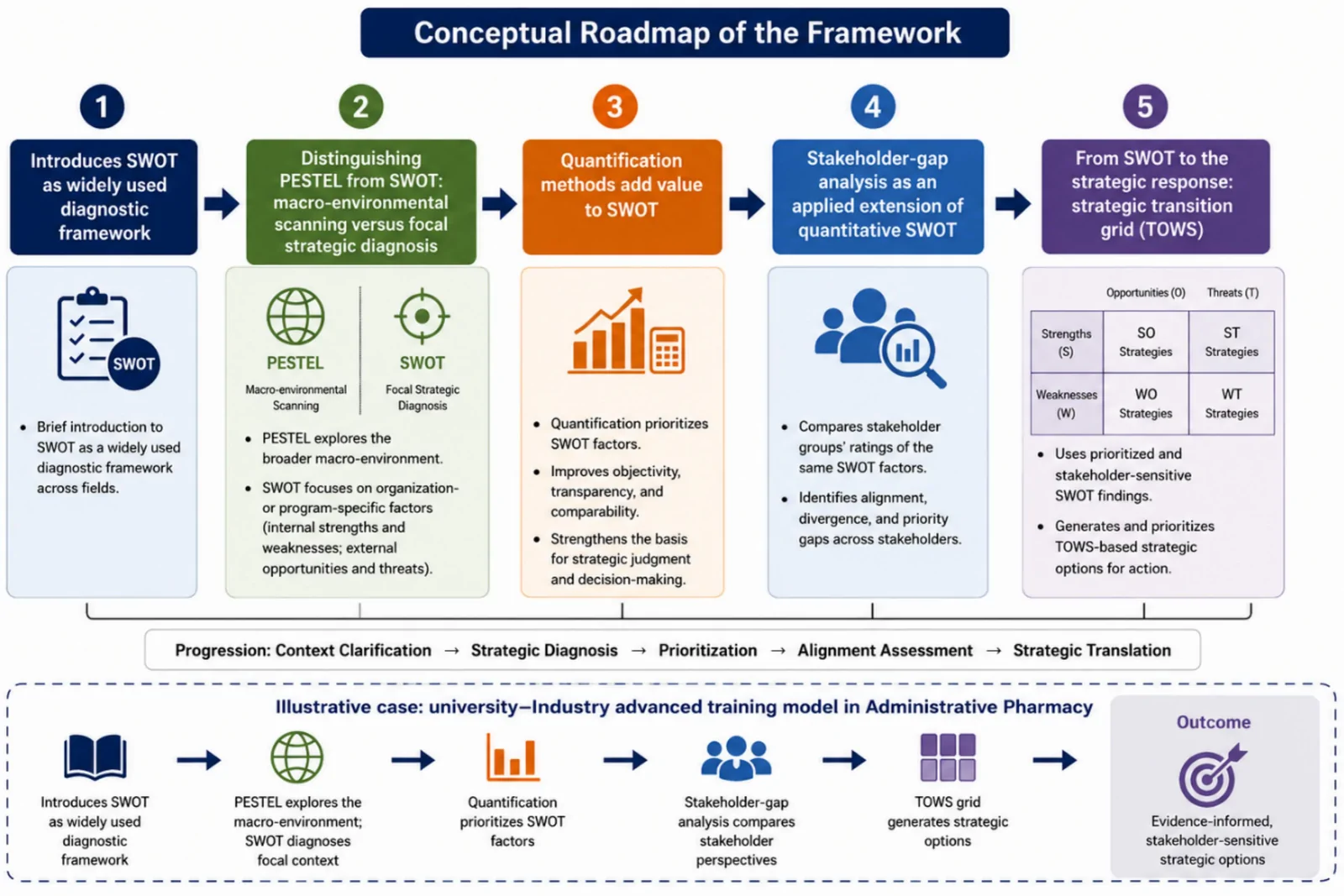
Conceptual roadmap of the framework from descriptive SWOT to quantitative SWOT in pharmacy strategy. SWOT, Strengths, Weaknesses, Opportunities, and Threats; PESTEL, Political, Economic, Social, Technological, Environmental, and Legal; TOWS, Threats, Opportunities, Weaknesses, and Strengths; SO, strengths–opportunities; WO, weaknesses–opportunities; ST, strengths–threats; WT, weaknesses–threats.

The framework advanced in this paper follows a five-stage logic that moves from **focal strategic diagnosis** to measurable strategic response (Fig. 1). First, SWOT is introduced as a widely used diagnostic tool.^16^, ^17^, ^22^ Second, PESTEL is distinguished from SWOT by separating macro-environmental scanning from focal organization- or program-specific strategic diagnosis.^23^, ^24^, ^25^ Third, quantification methods are presented as a way to improve the prioritization, transparency, and decision relevance of SWOT factors.^26^, ^27^, ^28^ Fourth, stakeholder-gap analysis is used to examine whether different stakeholder groups assign similar or divergent importance to the same SWOT factors.^20^, ^21^ Fifth, selected quantified and stakeholder-sensitive findings are translated into TOWS-based strategic options.^29^, ^30^

Together, these stages position quantitative SWOT as **a reproducible decision-support extension** of descriptive SWOT: PESTEL clarifies context, SWOT structures focal content, quantification clarifies priority, stakeholder-gap analysis clarifies alignment or separation, and the TOWS matrix supports strategic response. As shown in the lower panel of Fig. 1, the illustrative university–industry advanced training application follows the same sequence to demonstrate how the framework can be operationalized in a real pharmacy-strategy context.

Despite the broad use of descriptive SWOT in pharmacy, comparatively few pharmacy applications have operationalized SWOT as a quantified, stakeholder-sensitive, and action-linked decision-support process.^18^, ^19^, ^31^, ^32^ The novelty of the proposed framework lies in adapting quantitative SWOT logic from wider strategic-management and decision-science literature and integrating it with PESTEL contextual separation, stakeholder-gap analysis, and TOWS-based strategic translation into a single pharmacy-oriented pathway. Rather than treating quantitative SWOT as numerical scoring alone, the framework positions it as a structured process for moving from focal strategic diagnosis to measurable, stakeholder-sensitive strategic response.

### 2.1 SWOT origins, pharmacy use

*SWOT* evolved from mid-twentieth-century corporate planning and the earlier SOFT framework (*Satisfactory;* opening *Opportunities;* fixing *Faults;* or thwarting *Threats).*^16^ As a strategic assessment framework, SWOT classifies factors into strengths and weaknesses, which are internal to the focal organization or program, and opportunities and threats, which are external conditions that may support or constrain future performance. Its purpose is to support structured organizational reflection and strategic planning rather than to produce prioritization automatically.^18^, ^22^

In pharmacy sectors and policy-relevant pharmacy contexts, SWOT analysis has been applied across a wide range of practice, education, and industry settings. In hospital and community pharmacy, SWOT assessments have been used to examine service quality, patient satisfaction, infrastructure, pharmaceutical-care delivery, and opportunities for sustainable service development.^31^, ^33^, ^34^ In clinical pharmacy, SWOT has supported reflection on professional autonomy, interprofessional collaboration, and service-development barriers.^32^ In pharmacy education, it has been used to assess curriculum alignment, clinical-skill development, e-learning readiness, and responsiveness to labor-market expectations.^35^, ^36^, ^37^, ^38^ In pharmaceutical distribution, online pharmacy, and supply-chain contexts, SWOT has supported strategic reflection on competitiveness, logistics, digital transformation, and market adaptation.^19^, ^39^, ^40^

These applications demonstrate the breadth and practical relevance of SWOT across the pharmacy landscape. However, they also reveal the methodological gap addressed in this paper: SWOT is still frequently used as a descriptive mapping exercise rather than as an analytically structured decision-support method.^17^, ^22^ Descriptive SWOT may leave factors unranked and unweighted, limiting its ability to distinguish strategically decisive issues from those that are merely listed.^26^ This limitation becomes more important in multi-stakeholder pharmacy contexts, where different actors may assign different levels of importance to the same factor, as shown in recent stakeholder-based SWOT work in community pharmacy.^18^ Participatory and quantitative SWOT approaches can reduce arbitrariness, make priorities more explicit, and reveal stakeholder differences.^20^, ^21^ Accordingly, descriptive SWOT should be strengthened with quantitative and stakeholder-sensitive procedures that improve prioritization, analytical transparency, and strategic usefulness.

### 2.2 Distinguishing PESTEL from SWOT

A significant challenge involves the conceptual overlap between SWOT and PESTEL analysis. PESTEL is a macro-environmental scanning framework used to examine external forces that shape the strategic context in which organizations operate, including political, economic, social, technological, environmental, and legal conditions. In pharmaceutical and healthcare contexts, PESTEL has been used to assess how regulatory requirements, market conditions, technological change, social expectations, environmental pressures, and legal frameworks influence organizational strategy and system transformation.^23^, ^24^

Although the two frameworks are often used together, they do not serve the same analytical function. PESTEL is designed to scan the broader macro-environment that shape the operating environment of all actors within a sector or market. SWOT, by contrast, should remain specific to the focal organization, program, or system, and should explain the extent to which there is a fit between the organization and its environment. SWOT role is not merely to restate macro-environmental conditions, but to interpret how those conditions interact with the focal unit's internal capacities, vulnerabilities, and strategic position. When this distinction is ignored, SWOT can become overly generic, duplicative of PESTEL, and insufficiently tailored to the organization under study.^25^, ^41^, ^42^

In practical terms, a PESTEL analysis conducted in a given sector may yield broadly similar external conditions for multiple institutions operating in the same context, whereas each institution's SWOT profile should differ according to its resources, capabilities, constraints, and strategic responses.

For example, national demand for employability-oriented pharmacy education is better treated as a PESTEL-level condition because it reflects a broader social and economic factor affecting all pharmacy schools in the same sector. However, each institution's SWOT profile should differ according to its own resources and constraints: a school with an established industry-supervisor network may classify that network as a strength, whereas another school without such partnerships may identify limited partnership capacity as a weakness. Sector-wide conditions that apply similarly to multiple competitors should therefore remain within PESTEL rather than being transferred directly into SWOT, which should remain institution- or program-specific. Maintaining this distinction prevents SWOT from becoming a generic restatement of external context and preserves its value as a focal strategic assessment.

Basic questions and pharmacy-related examples that can help leaders, managers, and educators design SWOT or PESTEL analyses for their institutions, programs, services, or products are presented in Table 1.

**Table 1:** Distinguishing Descriptive SWOT and PESTEL.

Tool	Category	Pharmacy program/service analytical question⁎
**SWOT (Internal)**	**Strengths (S)**	What makes the pharmacy program or service better than comparable alternatives? Consider internal capabilities, resources, achievements, partnerships, expertise, distinctive competencies, stakeholder recognition, readily available advantages, or features that are difficult for others to replicate.
	**Weaknesses (W)**	What reduces the performance, credibility, consistency, or scalability of the pharmacy program or service? Consider missing competencies, resource gaps, coordination problems, service complaints, avoided issues, limited standardization, training needs, unmet objectives, or areas where comparable alternatives perform better.
**SWOT (External-Specific)**	**Opportunities (O)**	What favorable external conditions could the pharmacy program or service realistically use to enhance success or create value? Consider unmet sector needs, overlooked gaps, strategic contacts, new partners, emerging career pathways, digital tools, patient-service needs, missed but still available opportunities, or problems the program or service could help solve.
	**Threats (T)**	What external conditions could undermine the pharmacy program or service's feasibility, sustainability, or strategic position? Consider competing programs or services, rising competition, regulatory change, technology shifts, rising costs, workforce saturation, partner fatigue, market expectations, external obstacles, or internal weaknesses that could become future threats.
**PESTEL (Macro-External)**	**Political**	How do political conditions shape the broader context of the pharmacy program or service? Consider national health policies, professional governance, pharmacy-workforce planning, public-sector reforms, health-system priorities, or policy support for education, practice, and service innovation.
	**Economic**	How do economic conditions shape the broader context of the pharmacy program or service? Consider medicine-pricing and insurance systems, reimbursement conditions, unemployment, funding availability, purchasing power, training costs, pharmaceutical-market pressures, or economic incentives for employers and partners.
	**Social**	How do social conditions shape the broader context of the pharmacy program or service? Consider patient expectations, demographic change, public trust, professional role expansion, workforce culture, graduate expectations, employer expectations, or changing perceptions of pharmacy careers and services.
	**Technological**	How do technological conditions shape the broader context of the pharmacy program or service? Consider digital pharmacy systems, electronic health records, Interaction platforms, telepharmacy, online education, automation, data systems, digital communication, or technology-supported training and service delivery.
	**Environmental**	How do environmental conditions shape the broader context of the pharmacy program or service? Consider sustainability expectations, waste-management policies, environmental requirements, supply-chain pressures, resource-use concerns, green pharmacy initiatives, or climate-related disruptions affecting pharmacy systems.
	**Legal**	How do legal conditions shape the broader context of the pharmacy program or service? Consider pharmacy laws, licensing requirements, accreditation standards, medicine-promotion rules, data-protection obligations, labor regulations, professional liability, or sector-wide compliance requirements.

⁎ If the focal unit of analysis is a pharmacy product, brand, or marketing plan rather than a program or service, the term “pharmacy program or service” can be replaced with the relevant product, brand, or marketing-plan name, as appropriate.

### 2.3 Quantification methods add value to SWOT

In many real decision contexts, especially in pharmacy systems, not all strengths, weaknesses, opportunities, and threats are equally important.^26^ Some are marginal, some are decisive, and some are particularly important to specific stakeholder groups rather than to the system.^26^, ^43^ If all factors are treated as qualitatively equivalent, strategic prioritization becomes weak, budget allocation becomes diffuse, and decision-making remains vulnerable to impressionistic judgment.^43^ This is why quantification becomes important.^44^, ^45^

Quantitative SWOT can be implemented with different levels of analytical intensity, ranging from simple stakeholder rating to more advanced weighting, network-based, and uncertainty-sensitive methods. This progression should be understood as a scalable methodological pathway rather than as a hierarchy of universally superior methods: baseline rating or ranking may be sufficient for exploratory prioritization, whereas more complex approaches can be selected when context requires formal weighting, consistency checking, interdependence modeling, or uncertainty-sensitive prioritization management. The main quantitative extensions of SWOT, their methodological position, analytical logic, suitable use, and equations are summarized in Table 2.

**Table 2:** The main quantitative extension methods of SWOT, their levels, analytical logic, decision contexts.

Methodological position	Primary Output	Strategic Question Answered	Method	Core Analytical Logic	Decision Context / Suitable Use	Equation	Terms in the Equation
Baseline quantitative application†	Rank order or summary score	What appears most important?	Ordinal ranking / scoring	Stakeholders score or rank SWOT factors on a common scale; factors are then summarized and ordered to identify relative priority and support comparison across respondents or stakeholder groups.	Exploratory SWOT quantification, stakeholder workshops, early-stage program or service review, and rapid prioritization when formal weighting is not required.	x̄_i = (sum of x_ir from *r* = 1 to n) / n	x_ir = score of factor i by respondent r; n = number of respondents; x̄_i = mean score of factor i
Level 1	Composite rate scores (RS)	What is strategically decisive?	Composite rate scores, such as (Magnitude × Importance)	Each SWOT factor is scored on two or more predefined dimensions, commonly magnitude and strategic importance, then combined into a composite priority score.	Routine organizational planning, service improvement, operational prioritization, and settings where decision-makers need more discrimination than simple ranking.	RS_i_ = M_i_ × I_i_	RS_i = composite rate score of factors i; M_i = magnitude I_i = importance
Level 2	Eigenvector weights and consistency ratio	What is relatively most important across stakeholders?	AHP-SWOT (A'WOT)	Pairwise comparisons are used to derive relative weights for SWOT factors; consistency indices are calculated to assess the reliability of the judgment structure.	Formal prioritization, multi-stakeholder decision-making, resource-allocation decisions, and settings requiring defensible weighting of competing strategic factors.	A w = λ_max_ w; CI = (λmax − n)/(n − 1); CR = CI/RI	A = pairwise comparison matrix. w = priority weight vector; lambda_max = maximum eigenvalue. n = matrix order CI = consistency index RI = random index CR = consistency ratio
Level 3	Network-based priority weights	How do interdependencies shift priorities?	ANP-SWOT	Dependencies and feedback among SWOT factors or clusters are modeled in a network structure, allowing priorities to reflect interrelationships rather than independent factor effects only.	Complex systems where policy, workforce, infrastructure, financing, regulation, technology, and implementation factors interact dynamically.	W* = limit of W^k as k approaches infinity	W = weighted supermatrix; W^k = supermatrix raised to power k; W* = limit supermatrix containing stable priorities
Level 4	Fuzzy weights / defuzzified crisp priorities	What remains important under uncertainty?	Fuzzy SWOT / FAHP / SWOT-PIPRECIA	Judgments are expressed using fuzzy, linguistic, or uncertainty-sensitive values, then converted into usable priority estimates for decision-making.	High-uncertainty contexts, expert-based planning, policy-sensitive decisions, and situations where precise numerical judgments are difficult or premature.	a ∼ = (l, m, u); BNP = (l + m + u) / 3	a ∼ = triangular fuzzy number l = lower bound m = most plausible value u = upper bound BNP = best non-fuzzy performance
Complementary Diagnostic Tool	Strategic vector or strategic posture	What is our overall strategic posture?	SWOT Polar Coordinate / Strategic Vector Model*	Aggregated strength, weakness, opportunity, and threat scores are converted into a directional and strength indicator to summarize overall strategic posture rather than individual factor priority.	Macro-level strategic posture diagnosis after SWOT scores or weights have been generated; useful for visual positioning and high-level strategic interpretation.	θ theta = arctan(Y / X); X = (S − W) / 4; Y = (O − T) / 4; rho = U / (U + V); U = S × O; V = W × T	theta = strategic azimuth/direction; X,Y = centre-of-gravity coordinates; S = strengths intensity; W = weaknesses intensity; O = opportunities intensity; T = threats intensity; U = positive strategic intensity; V = negative strategic intensity; rho = strategic strength coefficient

† The illustrative empirical application in this paper used the baseline ordinal rating/scoring approach.

- Levels 1 to 4 are presented as scalable methodological extensions of increasing analytical intensity.

*The SWOT polar-coordinate model is presented as a complementary diagnostic tool for overall strategic posture rather than as a factor-prioritization level.

- Abbreviations: **SWOT**, strengths–weaknesses–opportunities–threats; **AHP**, Analytic Hierarchy Process; **A'WOT**, AHP integrated with SWOT; **ANP**, Analytic Network Process; **FAHP**, Fuzzy Analytic Hierarchy Process; **PIPRECIA**, Pivot Pairwise Relative Criteria Importance Assessment; **BNP**, Best Non-fuzzy Performance; **RS**, Rate Score.

As shown in Table 2, the baseline quantitative application involves ordinal ranking or Likert-type rating of SWOT factors. This approach does not produce formal weights, but it is useful for rapid priority identification and for exposing differences in stakeholder perceptions. Its main strength lies in simplicity and feasibility, particularly in exploratory settings or workshop-based strategic exercises. A practical extension of this approach is the weighted SWOT matrix, presented in Table 2 as Level 1. This approach often uses composite scoring, in which each factor is rated for magnitude (M), meaning the extent to which it currently influences organizational performance or risk exposure, and importance (I), meaning its strategic criticality for achieving organizational objectives. These two scores are then multiplied to produce a composite rate scores.^27^, ^28^, ^45^ This method remains accessible to managers and practitioners in pharmacy related fields while generating more differentiated prioritization than simple ranking.^26^

When stronger methodological justification is needed, more advanced approaches can be used. Level 2, Analytic Hierarchy Process integrated with SWOT (AHP–SWOT or A'WOT), applies pairwise comparison and eigenvector derivation to generate relative weights for strategic factors and is especially useful in multi-stakeholder contexts requiring structured prioritization and consistency checking.^44^, ^46^ Level 3, Analytic Network Process integrated with SWOT (ANP–SWOT), extends this logic further by allowing for interdependence and feedback among factors, which is especially relevant in complex systems where policy, workforce, service design, financing, and implementation interact dynamically.^47^, ^48^ Under conditions of greater ambiguity or expert uncertainty, **Level 4** includes operations as fuzzy hybrids,^49^ fuzzy AHP,^50^, ^51^ and related methods such as SWOT–PIPRECIA (Pivot Pairwise Relative Criteria Importance Assessment) can provide more robust weighting under imprecise or linguistically expressed judgments.^52^ Finally, for organizations seeking a macro diagnostic of overall strategic posture rather than factor-level prioritization, the SWOT polar coordinate model can be used as a complementary diagnostic tool, rather than as another prioritization level, because it summarizes the net direction and strength of the strategic vector.^52^

Not every strategic problem requires mathematically advanced modeling.^28^ In many pharmacy settings, the optimal approach is the minimum sufficient method that provides transparent prioritization without unnecessary complex analytical burden.^26^

### 2.4 Stakeholder-gap analysis, an extension of quantitative SWOT

Quantitative SWOT is especially valuable when the purpose of the analysis is not only to enumerate strategic factors, but also to determine whether different stakeholder groups assign similar or divergent importance to those factors. Quantitative SWOT also enables a further analytical application: the assessment of stakeholder-priority gaps.

In pharmacy strategy, this application is especially relevant because implementation commonly depends on alignment across actors with different roles, incentives, and evaluative criteria. By converting stakeholder judgments into comparable ranks, scores, weights, or composite priorities, quantitative SWOT makes these differences explicit and therefore allows inter-stakeholder comparison as an additional layer of analysis. Recent methodological and applied work shows that participatory and weighted SWOT approaches can reduce arbitrariness, strengthen prioritization, and reveal differences in emphasis across stakeholder groups that would remain insufficiently visible under descriptive SWOT alone.^20^, ^21^, ^53^, ^54^ Accordingly, stakeholder-gap analysis should be viewed as a distinct applied extension of quantitative SWOT, particularly in multi-actor settings where strategy must be negotiated rather than assumed.

### 2.5 From SWOT to the strategic response

The preceding sections establish the analytical sequence of the framework: SWOT provides the focal diagnostic structure, PESTEL separates broader macro-environmental conditions from organization- or program-specific analysis, quantitative methods clarify the relative priority of SWOT factors, and stakeholder-gap analysis identifies areas of alignment or divergence across stakeholder groups. Consistent with the conceptual roadmap presented in Fig. 1, the final step is strategic translation, where TOWS matrix^29^, ^30^, ^55^, ^56^ provides the methodological bridge from prioritized and stakeholder-sensitive diagnosis to strategic response.

**Fig. 2:**
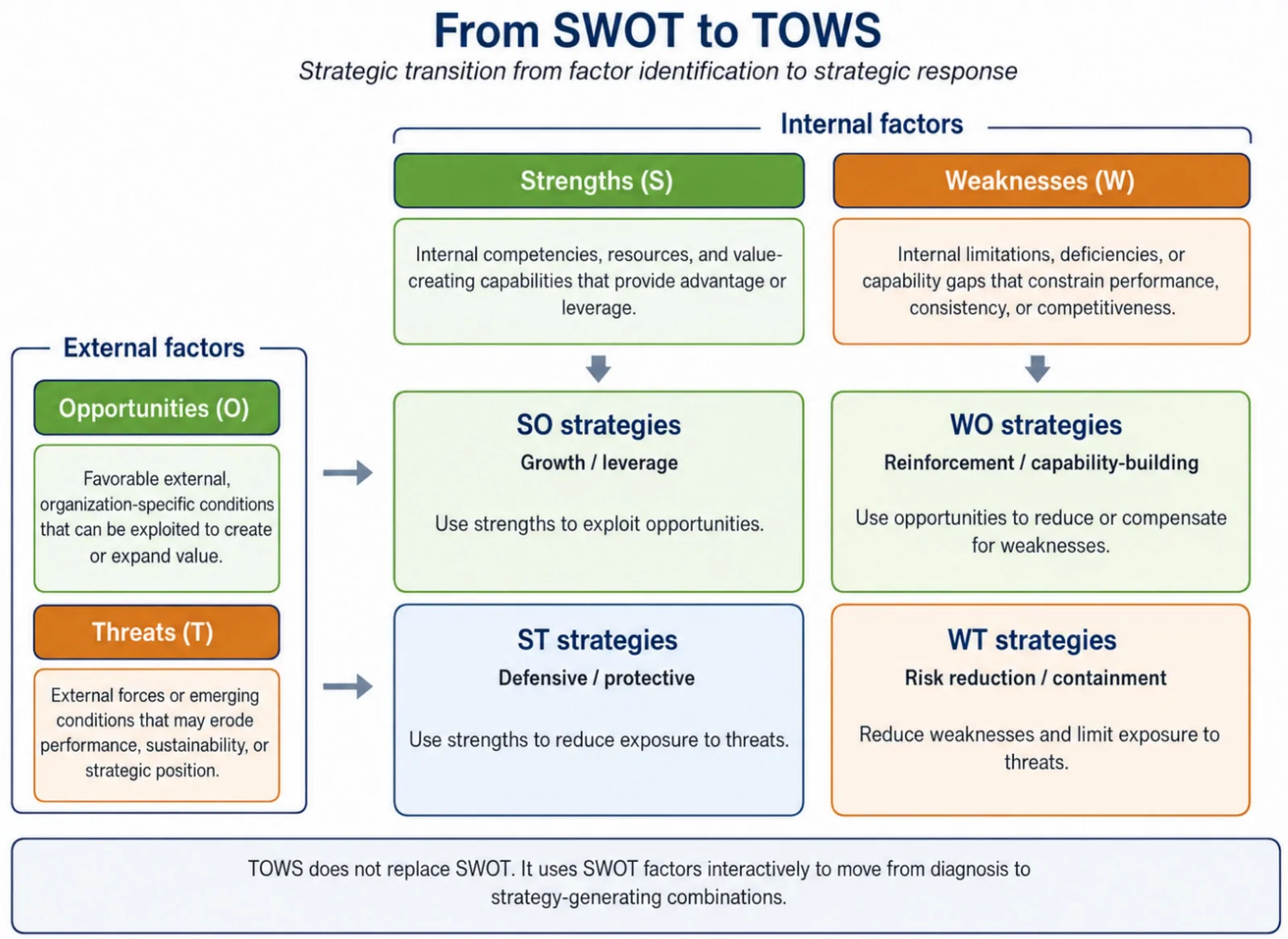
From SWOT to TOWS: strategic transition from factor identification to strategic response. The figure shows how internal strengths (S) and weaknesses (W) are combined with external opportunities (O) and threats (T) to generate four strategic postures: SO, WO, ST, and WT. - SWOT, Strengths, Weaknesses, Opportunities, and Threats. - TOWS, Threats, Opportunities, Weaknesses, and Strengths. - SO, strengths–opportunities; WO, weaknesses–opportunities; ST, strengths–threats; WT, weaknesses–threats.

As shown in Fig. 2, the TOWS matrix intersects internal strengths and weaknesses with external opportunities and threats to generate four strategic postures^29^, ^30^, ^55^, ^56^:

• Strengths–opportunities (SO) strategies, use internal strengths to exploit external opportunities and are typically growth- or leverage-oriented.
• Weaknesses–opportunities (WO) strategies, use external opportunities to reduce or compensate for internal weaknesses and are therefore reinforcement- or capability-building strategies.
• Strengths–threats (ST) strategies, use internal strengths to reduce exposure to external threats and are defensive or protective strategies.
• Weaknesses–threats (WT) strategies minimize internal weaknesses while reducing exposure to external threats. In practice, WT strategies are used when internal fragility and external pressure interact in ways that may undermine feasibility, sustainability, or strategic position. Responses may include containment, restructuring, risk reduction, tighter coordination, selective redesign, or withdrawal from high-vulnerability areas where necessary.

This distinction is important because the value of SWOT lies not only in identifying strategic factors, but also in supporting a structured transition from diagnosis to action. TOWS matrix provides that transition by reorganizing SWOT factors into strategy-generating combinations rather than leaving them as separate lists.

## 3 Illustrative empirical application of the framework

### 3.1 Case and background

The illustrative application briefly demonstrates how the quantitative SWOT framework can be operationalized through a real university–industry advanced training model in Administrative Pharmacy at the Hashemite University. This application is used to demonstrate methodological transferability, not to provide an exhaustive local program evaluation. To keep the empirical section proportionate to the paper's conceptual-methodological aim, Supplementary Table S1 presents the case profile and the most relevant contextual features of Jordanian pharmacy education, labor-market expectations, and regulated professional training.

### 3.2 Method

The application is presented as a worked example of the framework's reproducible steps rather than as an outcome evaluation of the training program. The empirical application used a mixed-methods, cross-sectional, stakeholder-based design. Supplementary Table S2 summarizes the operational details of the application, including the setting, stakeholder groups, documentary sources, factor-development process, quantification approach, statistical analysis, stakeholder comparison, strategic translation, and ethical approval.

In practical application, we suggest that quantitative SWOT analysis typically involves three distinct roles. The analytical lead structures the process, drafts and refines the strategic statements, organizes the quantitative method, and interprets the resulting profile. Stakeholders contribute contextual input and rating judgments according to their institutional positions and priorities. The decision owner then uses the resulting evidence to approve priorities, select strategy direction, or guide implementation. Quantitative SWOT should therefore be understood as an analytically led, stakeholder-informed, and decision-oriented process rather than as an unstructured aggregation of opinions alone. To improve reproducibility, the framework requires analytical lead to define the focal unit of analysis, the source of SWOT statements, the stakeholder groups, the rating or weighting method, the comparison procedure, the gap-classification rule, and the logic used to select factors for strategic translation.

Because the empirical component was intended to illustrate the proposed roadmap rather than provide an exhaustive focal case evaluation, the analysis used the baseline quantitative SWOT rating/scoring approach described earlier, rather than the higher-intensity quantitative extensions involving formal weighting, interdependence modeling, or uncertainty-sensitive prioritization. Accordingly, the empirical application followed the conceptual sequence of the framework: the focal training model was first defined using SWOT as the diagnostic frame; PESTEL was then used to distinguish broader macro-environmental conditions from program-specific SWOT factors; the finalized SWOT profile was quantified through baseline stakeholder ratings; and the results were extended through stakeholder-gap analysis and illustrative TOWS-based strategic translation.

Candidate PESTEL and SWOT statements were generated from program documents, implementation records, and stakeholder-group sessions. PESTEL was used to map the macro-environmental context, while SWOT was restricted to factors specific to the Administrative Pharmacy training model. Final SWOT statements were refined, de-duplicated, classified, and circulated through a structured Google Form to academics, partner managers or industry representatives, and students or trainees.

Respondents rated each factor on a 5-point ordinal importance scale, with higher scores indicating greater perceived importance. Mean (SD) values were reported only as descriptive prioritization scores to facilitate comparison across multiple SWOT factors and stakeholder groups; inferential interpretation was based on rank-based nonparametric testing. Given the ordinal scale and the small, unequal stakeholder groups, inter-group differences were examined using the Kruskal–Wallis test, with Bonferroni-adjusted pairwise comparisons where appropriate. The empirical findings were interpreted as illustrative stakeholder-prioritization patterns rather than statistically generalizable estimates.

### 3.3 Results

#### 3.3.1 Descriptive SWOT profile of the focal program

A descriptive SWOT profile was developed to identify the program's internal strengths and weaknesses and its external opportunities and threats (Table 3). Statements were refined to remove overlap and retain distinct factors suitable for later stakeholder rating. Overall, the profile highlighted first-mover structural distinctiveness and employability relevance as key strengths; coordination, staffing, standardization, and scalability constraints as main weaknesses; partner expansion and labor-market alignment as major opportunities; and competitive imitation, variable partner commitment, and uneven training exposure as leading threats. Codes were assigned to each SWOT factor to enable cross-reference across the descriptive SWOT profile, baseline quantitative ratings, stakeholder-gap analysis, and TOWS-based strategic translation.

**Table 3:** Descriptive SWOT profile of the focal program, with concise internal present factors classified as strengths and weaknesses, and external forward-looking factors classified as opportunities and threats.

Code	Strength	Code	Weakness
S1	The program provides structured field-based training beyond routine placement.	W1	The program has a high coordination burden across partners and sites.
S2	The program represents a first-mover and uniquely tailored Administrative Pharmacy training model within the Jordanian pharmacy education context.	W2	The program depends heavily on one academic coordinator for follow-up.
S3	The program includes three differentiated training streams.	W3	Implementation and monitoring are not fully standardized across sites.
S4	The program uses a joint academic–industry partnership model with Training of Trainer induction, tailored preparation, and student-specific training maps.	W4	The two-day field format may limit training depth.
S5	The program benefits from leadership with direct industry experience.	W5	The program has limited dedicated funding for partner and student training costs.
S6	The curriculum provides two protected training days per week.	W6	The program may be difficult to scale because of transport, location, and mobility constraints.
**Code**	**Opportunity**	**Code**	**Threat**
O1	The program can benefit from growing demand for non-traditional pharmacy careers.	T1	Other universities may replicate the model and compete for partner slots.
O2	The program can expand through additional strategic partners.	T2	Labor-market pressure may increase expectations for visible employability outcomes.
O3	The program can engage companies that benefit from student trainees.	T3	Conventional community- and hospital-based training expectations may limit support for this model.
O4	The program can use digital tools, CRM systems, and webinars to strengthen training.	T4	Variation across partner practices may create unequal training exposure.
O5	The program can improve university-to-work transition pathways.	T5	Because training is not the partners' primary mission, student activities may be seen as a burden.
O6	The program can strengthen the visibility of Administrative Pharmacy.	T6	The limited weekly time structure may reduce perceived business value for partners.

Abbreviations: S = strength; W = weakness; O = opportunity; T = threat.

Numbers (codes) indicate item identifiers within each SWOT category and are used for cross-reference across subsequent analyses. They do not represent ranking, importance, priority, or statistical weight.

#### 3.3.2 Macro-contextual separation (PESTEL analysis)

A PESTEL analysis (Supplementary Table S3). was used to distinguish broader macro-environmental conditions from the program-specific SWOT profile. Rather than duplicating the SWOT analysis, this step clarified the wider political, economic, social, technological, environmental, legal, and sectoral conditions shaping pharmacy education, labor-market expectations, and professional practice in Jordan. The resulting contextual map helped preserve the specificity of the focal SWOT profile by separating sector-level conditions from factors directly linked to the administrative pharmacy training model.

#### 3.3.3 Baseline quantitative SWOT ratings

Baseline quantitative SWOT ratings are presented in Supplementary Table S4. This step moved the analysis beyond descriptive factor listing by showing how academics, partner managers or industry representatives, and students (trainees) prioritized the finalized SWOT factors on a common ordinal scale.

The baseline profile suggested broad agreement on the value of the joint academic–industry model (S4), industry-informed leadership (S5), and the program's university-to-work transition function (O5), with no statistically significant overall differences across stakeholder groups. More pronounced stakeholder separation appeared around factors linked to implementation and strategic positioning. Academics and students assigned higher priority than managers to the program's first-mover identity (S2; *P* = .011), differentiated training streams (S3; *P* = .001), protected two-day field structure (S6; *P* < .001), digital strengthening opportunities (O4; *P* = .003), and competitive-imitation threat (T1; *P* = .003). By contrast, managers placed greater emphasis on operational constraints, including training-depth concerns (W4; *P* = .039), scalability limitations (W6; *P* = .020). Descriptively, managers also gave higher ratings to variation across partner practices (T4) and the perceived business value of the weekly training structure (T6), although these differences were not statistically significant. These patterns informed the stakeholder-gap analysis presented in Table 4.

**Table 4:** Stakeholder-gap analysis of baseline quantitative SWOT: direction and strength of inter-stakeholder differences.

	SWOT factor	P	M vs A	M vs S	A vs S	Post hoc pattern (Bonferroni-adjusted)	Interpretive category
S1	Structured field-based training beyond routine placement	0.021	Lower	Lower	Slightly higher	M < S (0.086); M < A (0.072); A > S (1.0)	Diffuse gap
S2	First-mover and uniquely tailored Administrative Pharmacy model	0.011	Lower	Lower	Slightly higher	M < S (0.050); M < A (0.044); A > S (1.0)	Moderate gap
S3	Three differentiated training streams	0.001	Lower	Lower	Higher	M < S (0.028); M < A (0.003); A > S (1.0)	Strong gap
S4	Joint academic–industry partnership model with ToT and student-specific maps	0.053	Slightly lower	Slightly lower	Aligned	NS overall; post hoc not pursued	Non-significant descriptive alignment
S5	Leadership with direct industry experience	0.431	Slightly lower	Slightly lower	Aligned	NS overall; post hoc not pursued	Non-significant descriptive alignment
S6	Two protected training days per week	<0.001	Markedly lower	Markedly lower	Slightly higher	M < S (0.005); M < A (0.003); A > S (1.0)	Strong gap
W1	High coordination burden across partners and sites	0.001	Slightly lower	Higher	Higher	M > S (0.003); M < A (0.007); A > S (1.0)	Strong gap
W2	Dependence on one academic coordinator	0.001	Lower	Higher	Higher	M > S (0.003); M < A (0.004); A > S (1.0)	Strong gap
W3	Implementation and monitoring not fully standardized across sites	0.017	Slightly lower	Higher	Higher	M > S (0.034); M < A (0.048); A > S (1.0)	Strong gap
W4	Two-day field format may limit training depth	0.039	Higher	Higher	Aligned	M > S (1.0); M > A (0.070); A ≈ S (0.237)	Diffuse gap
W5	Limited dedicated funding for partner and student training costs	0.325	Slightly lower	Slightly higher	Higher	NS overall; post hoc not pursued	Non-significant descriptive alignment
W6	Difficult to scale because of transport, location, and mobility constraints	0.02	Higher	Higher	Lower	M > S (0.948); M > A (0.029); A < S (0.244)	Moderate gap
O1	Growing demand for non-traditional pharmacy careers	0.04	Lower	Lower	Slightly higher	M < S (0.187); M < A (0.090); A > S (1.0)	Diffuse gap
O2	Expansion through additional strategic partners	0.012	Lower	Lower	Slightly higher	M < S (0.117); M < A (0.026); A > S (1.0)	Moderate gap
O3	Engage companies that benefit from student trainees	0.002	Markedly lower	Lower	Slightly higher	M < S (0.025); M < A (0.009); A > S (1.0)	Strong gap
O4	Use digital tools, CRM systems, and webinars to strengthen training	0.003	Markedly lower	Markedly lower	Slightly higher	M < S (0.025); M < A (0.013); A > S (1.0)	Strong gap
O5	Improve university-to-work transition pathways	0.142	Slightly lower	Slightly lower	Slightly higher	NS overall; post hoc not pursued	Non-significant descriptive alignment
O6	Strengthen the visibility of Administrative Pharmacy	0.088	Slightly lower	Slightly lower	Slightly higher	NS overall; post hoc not pursued	Non-significant descriptive alignment
T1	Other universities may replicate the model and compete for partner slots	0.003	Lower	Lower	Slightly higher	M < S (0.019); M < A (0.018); A > S (1.0)	Strong gap
T2	Labor-market pressure may increase expectations for visible employability outcomes	0.092	Lower	Lower	Slightly higher	NS overall; post hoc not pursued	Non-significant descriptive spread
T3	Conventional community- and hospital-based training expectations may limit support for this model	0.162	Lower	Slightly higher	Higher	NS overall; post hoc not pursued	Non-significant descriptive spread
T4	Variation across partner practices may create unequal training exposure	0.106	Higher	Higher	Lower	NS overall; post hoc not pursued	Non-significant descriptive spread
T5	Because training is not the partners' primary mission, student activities may be seen as a burden	0.412	Slightly higher	Higher	Slightly higher	NS overall; post hoc not pursued	Non-significant descriptive alignment
T6	The limited weekly time structure may reduce perceived business value for partners	0.159	Higher	Higher	Lower	NS overall; post hoc not pursued	Non-significant descriptive spread

-Abbreviations: M = Managers; A = Academics; S = Students. *p*-values indicate Kruskal–Wallis overall three-group difference for each SWOT factor.

-Direction in the M vs A, M vs S, and A vs S columns was assigned from the stakeholder-group means in Supplementary Table S4.

-The Post hoc pattern column reports the corresponding Bonferroni-adjusted pairwise *p*-values from SPSS; therefore, entries such as M < A (0.044) indicate that managers rated the item descriptively lower than academics, with an adjusted pairwise *p*-value of 0.044.

-Strong gap = statistically significant overall Kruskal–Wallis result with two Bonferroni-adjusted pairwise contrasts <0.05.

-Moderate gap = statistically significant overall Kruskal–Wallis result with one Bonferroni-adjusted pairwise contrast <0.05.

-Diffuse gap = statistically significant overall Kruskal–Wallis result without Bonferroni-adjusted pairwise contrasts <0.05.

-Non-significant descriptive alignment = overall Kruskal–Wallis *P* ≥ .05 with relatively small spread across stakeholder-group means, indicating broad descriptive alignment but not a statistically supported stakeholder gap.

-Non-significant descriptive spread = overall Kruskal–Wallis *P* ≥ .05 with more visible spread across stakeholder-group mean scores, indicating non-significant but descriptively apparent separation, not a statistically supported stakeholder gap.

-For non-significant results, descriptive alignment or spread was determined from the observed spread of stakeholder-group mean scores: a maximum mean difference of <1.00 was treated as non-significant descriptive alignment, whereas a maximum mean difference of ≥1.00 was treated as non-significant descriptive spread. This criterion was used only for descriptive interpretation and to support the illustrative TOWS matrix translation; it was not treated as a separate test of significance.

#### 3.3.4 Stakeholder-gap analysis

A second question in quantitative SWOT is not only what stakeholders prioritize, but where statistically supported stakeholder gaps occur. Table 4 presents the stakeholder-gap analysis derived from the baseline ratings shown in Supplementary Table S4. This component is presented as an illustrative decision-support application of quantitative SWOT. Because the empirical example is intended to demonstrate the conceptual roadmap rather than provide a definitive focal case evaluation, the table distinguishes statistically supported stakeholder gaps from non-inferential descriptive patterns that may still inform illustrative TOWS matrix translation.

Statistically supported gaps were identified only when the overall Kruskal–Wallis test was significant. These gaps clustered around implementation structure, operational burden, and strategic positioning. Managers assigned lower priority than academics and/or students to the program's first-mover Administrative Pharmacy identity (S2), differentiated training streams (S3), the protected two-day field structure (S6), company-engagement opportunities (O3), digital strengthening opportunities (O4), and competitive-imitation threat (T1). In contrast, academics and managers gave higher priority than students to coordination burden (W1), single-coordinator dependence (W2), and limited standardization (W3), while managers gave higher priority than academics to scalability constraints (W6). Factors with significant overall differences but without Bonferroni-adjusted pairwise separation were retained as diffuse gaps. Non-significant results were not interpreted as stakeholder gaps; where retained, they were described only as non-inferential descriptive alignment or spread to support cautious decision-oriented interpretation.

#### 3.3.5 Translating quantified SWOT into strategic response

**Fig. 3:**
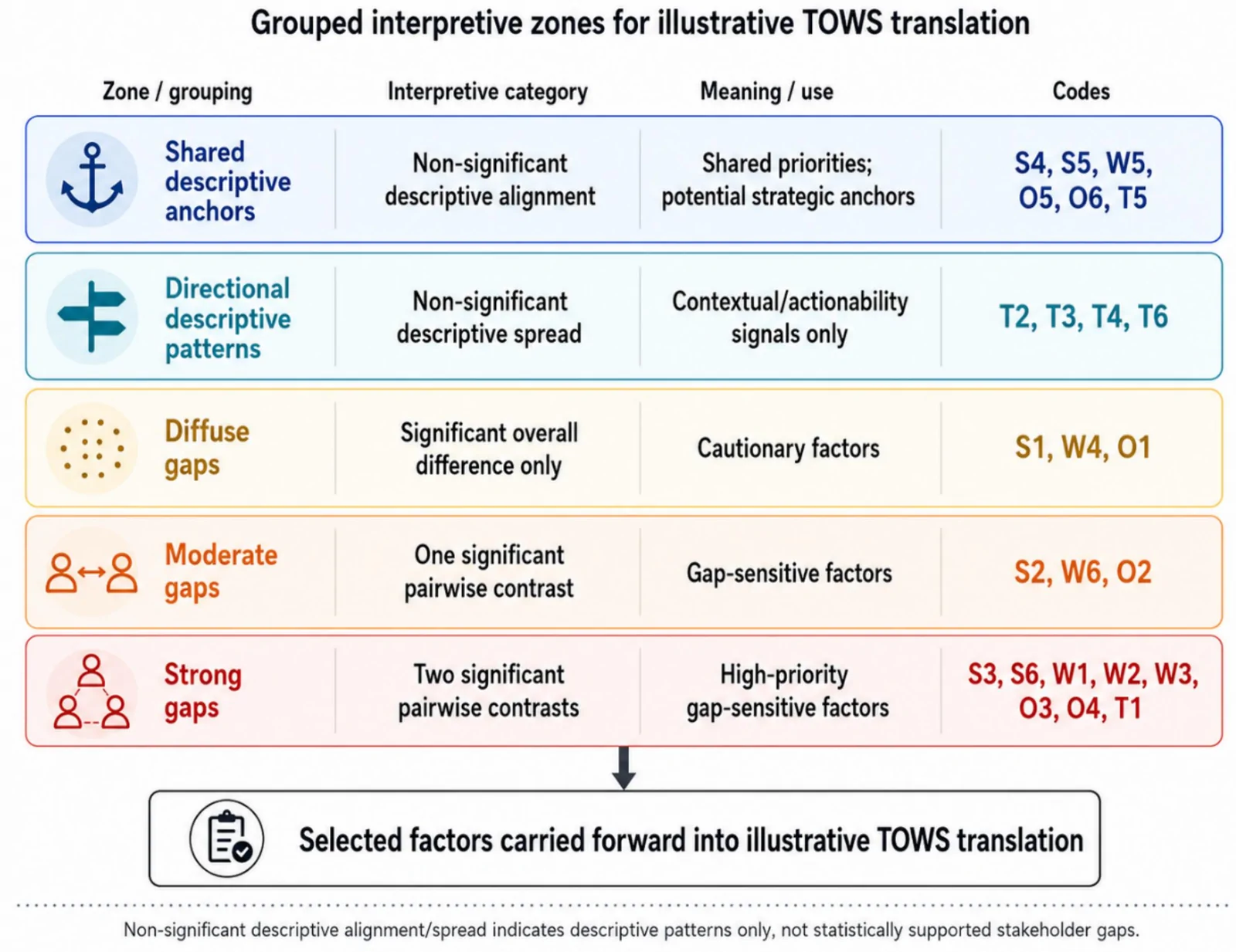
Grouped interpretive zones for illustrative TOWS matrix translation. The figure summarizes how quantified SWOT factors were grouped after stakeholder-gap analysis to guide selective TOWS-based strategic translation. Codes refer to the SWOT factors listed in Table 3: S = strength, W = weakness, O = opportunity, and T = threat; numbers indicate item identifiers within each SWOT category and do not represent rank, importance, priority, or statistical weight. Non-significant descriptive alignment and non-significant descriptive spread indicate descriptive patterns only and were not interpreted as statistically supported stakeholder gaps. Diffuse gap indicates a statistically significant overall Kruskal–Wallis result without Bonferroni-adjusted pairwise significance. Moderate gap indicates one Bonferroni-adjusted pairwise contrast <0.05. Strong gap indicates two Bonferroni-adjusted pairwise contrasts <0.05. SWOT = Strengths, Weaknesses, Opportunities, and Threats; TOWS = Threats, Opportunities, Weaknesses, and Strengths.

To support strategic translation, the stakeholder-gap profile was condensed into grouped zones of agreement and divergence rather than carried forward as a full item-by-item list (Fig. 3). Shared strategic anchors were defined as factors showing shared descriptive importance that could provide a relatively stable basis for strategy formulation. Gap-sensitive factors were defined as factors with statistically supported moderate or strong stakeholder separation, indicating issues where implementation may require closer attention to stakeholder priorities, feasibility, or buy-in.

Descriptive SWOT alone identified and classified the program's strengths, weaknesses, opportunities, and threats, but it did not show which factors were shared across stakeholders, which were stakeholder-sensitive, or which were most suitable for strategic translation. Thus, the comparison is not between two separate empirical studies, but between two analytical outputs from the same SWOT profile: descriptive SWOT provided classification, whereas the quantitative extension added prioritization, stakeholder sensitivity, and a transparent basis for selective TOWS translation. The grouped synthesis in Fig. 3 therefore served as an intermediate step between descriptive SWOT and TOWS matrix by separating shared descriptive anchors, non-significant descriptive patterns, and statistically supported stakeholder gaps.

On this basis, the illustrative TOWS matrix translation did not carry forward all quantified SWOT factors simultaneously. The subset was selected by the analytical lead to demonstrate strategic translation rather than to produce an exhaustive strategy plan for the focal program. Selection was guided by three criteria: shared descriptive importance, meaning factors that could serve as strategic anchors; stakeholder sensitivity, meaning factors with moderate or strong stakeholder gaps that could affect implementation, prioritization, or buy-in; and practical actionability, meaning factors that could be translated into feasible SO, ST, WT, or WO strategic responses. Table 5 shows these selection zones explicitly so that the basis for each TOWS option is transparent.

**Table 5:** Illustrative TOWS-based strategic options derived from quantified and stakeholder-sensitive SWOT findings.

TOWS Matrix/Strategic posture	Selection zone/criterion	Selected factor basis⁎	Quantitative justification	Illustrative strategic direction
**SO strategy***(growth / leverage)*	Shared descriptive importance / strategic anchors	S4 Structured academic–industry model; S5 Industry-informed leadership; O5 University-to-work transition; O6 Administrative Pharmacy visibility	Shared descriptive anchors; no statistically supported stakeholder gap. Selected because they provide a stable basis for growth-oriented translation.	Leverage the program's structured academic–industry model and industry-informed leadership to strengthen visibility and position the program more clearly as a credible university-to-work transition pathway in Administrative Pharmacy.
**ST strategy***(defensive / protection)*	Stakeholder sensitivity + practical actionability	S2 First-mover identity; S3 Differentiated training streams; T1 Competitive imitation; T4 Unequal site exposure	S2: lower rated by Managers; moderate gap. S3: lower rated by Managers; strong gap. T1: lower rated by Managers; strong gap. T4: non-significant descriptive spread retained only as a contextual/actionability factor.	Use the program's first-mover identity and differentiated training structure to protect strategic distinction, strengthen partner commitment, and reduce vulnerability to competitive imitation and uneven exposure across sites.
**WT strategy***(risk reduction / containment)*	Stakeholder sensitivity + practical actionability	W6 Scaling constraints; W1 Coordination burden; T5 Partner burden perception; T6 Limited partner business value	W6: greater rated by Managers; moderate gap. W1: greater rated by Academics/ Managers; strong gap. T5: non-significant descriptive alignment; T6: non-significant descriptive spread. T5 and T6 were retained as contextual/ actionability factors, not statistically supported gaps.	Reduce operational vulnerability by redesigning implementation logistics and partner engagement to address scaling constraints, lower perceived partner burden, and improve the practical value of the current weekly training structure.
**WO strategy***(reinforcement / capability-building)*	Stakeholder sensitivity + practical actionability	W1 Coordination burden; W2 Single-coordinator dependence; W3 Limited standardization; O2 Partner expansion; O4 Digital training support	W1–W3: greater rated by Academics/ Managers; strong gaps. O2: lower rated by Managers; moderate gap. O4: lower rated by Managers; strong gap Selected because they connect implementation weaknesses with actionable opportunities for reinforcement.	Use partner expansion and digital training support to reduce coordination burden, lessen dependence on a single academic coordinator, and improve implementation standardization across training sites.

⁎ Selected factors were drawn from three zones of the quantified profile: strategic anchors based on shared descriptive importance, gap-sensitive factors based on moderate or strong stakeholder separation, and practically actionable factors suitable for illustrative TOWS matrix translation. Non-significant descriptive alignment or spread was used only as contextual/actionability information and was not interpreted as a statistically supported stakeholder gap.

The resulting matrix in Table 5 illustrates how quantitative SWOT can inform strategic response more explicitly than descriptive SWOT alone. In the descriptive SWOT profile, the same factors were only listed under strengths, weaknesses, opportunities, and threats. After quantification and stakeholder-gap analysis, however, they could be interpreted as shared strategic anchors, statistically supported stakeholder-sensitive issues, or contextual/actionability factors. TOWS matrix translation then reorganized selected factors into growth, defensive, risk-reduction, and capability-building options. This comparison shows the added practical value of the proposed framework: it does not simply restate the SWOT list, but makes the transition from factor identification to prioritized, stakeholder-sensitive strategic response more transparent.

## 4 Discussion

This paper shows how quantitative SWOT can strengthen pharmacy strategic planning by moving SWOT from descriptive factor identification toward prioritization, stakeholder comparison, and TOWS-based strategic response. This contribution is important because descriptive SWOT is widely used and accessible, but its conventional use often leaves factors unranked, unweighted, and weakly connected to action.^17^, ^22^, ^53^ The main contribution is not that SWOT can be quantified for its own sake, but that quantification can make strategic diagnosis more discriminating, transparent, and decision-relevant in multi-stakeholder pharmacy contexts.

**The illustrative application demonstrated the value of this framework through the five-stage sequence introduced in the conceptual roadmap. First**, SWOT provided the focal diagnostic structure for identifying program-specific strengths, weaknesses, opportunities, and threats. Second, PESTEL separated broader macro-environmental conditions from the focal SWOT profile. This distinction is methodologically important because PESTEL is intended to clarify macro-environmental forces, whereas SWOT should remain specific to the focal organization, program, service, or policy.^23^, ^24^, ^25^ In the empirical application, the diversity of pharmacy employment pathways and the growing demand for non-traditional pharmacy careers represented broader contextual conditions, whereas the program's first-mover Administrative Pharmacy identity, differentiated training streams, and structured academic–industry model represented case-specific strategic factors.

Third, baseline quantification showed which SWOT factors were shared, prioritized, or stakeholder dependent. This moved the analysis beyond descriptive listing toward comparative strategic judgment. Quantitative and weighted SWOT approaches have been proposed to reduce arbitrariness, strengthen prioritization, and support more structured decision-making.^27^, ^28^, ^44^, ^45^, ^46^, ^47^, ^48^, ^49^, ^50^, ^51^ In the empirical application, managers differed from academics and students on several strategically important items, assigning lower weight to the program's first-mover identity, differentiated training streams, digital strengthening opportunities, and two-day field structure, while placing greater emphasis on coordination burden, single-coordinator dependence, limited standardization, and scalability constraints. Quantification therefore did not simply add numbers; it clarified which factors were broadly shared strategic anchors and which became more contested across stakeholder groups.

Fourth, stakeholder-gap analysis clarified where stakeholder priorities aligned or separated. This step was particularly important because pharmacy-related implementation often depends on actors with different institutional roles, incentives, and evaluative priorities. Participatory SWOT and stakeholder-sensitive decision approaches can make such differences more visible and reduce the risk that strategic planning reflects only the assumptions of one dominant group.^18^, ^20^, ^21^ The gap profile in the illustrative application showed that academic and student perspectives were more strongly oriented toward educational distinctiveness and employability value, whereas partner managers emphasized operational feasibility, scalability, and partner-side value.

Fifth, actionable strategic options were developed through TOWS matrix translation, whereby selected quantified and stakeholder-sensitive SWOT findings were combined into SO, WO, ST, and WT responses. TOWS matrix is useful because it does not merely repeat the SWOT list; it links internal and external factors to support strategy formulation through growth, reinforcement, defensive, and risk-reduction responses.^29^, ^30^, ^55^, ^56^ In the illustrative application, shared strengths and opportunities, including the structured academic–industry model, industry-informed leadership, and university-to-work transition potential, informed SO growth strategies focused on visibility and positioning. Weaknesses such as coordination burden, single-coordinator dependence, and limited standardization were paired with opportunities such as partner expansion and digital training support to inform WO reinforcement and capability-building strategies. Strengths related to first-mover identity and differentiated training streams were paired with threats such as competitive imitation and uneven site exposure to support ST defensive strategies. Finally, weaknesses and threats related to scalability, partner burden, and limited partner-side value informed WT risk-reduction strategies focused on implementation redesign and sustainability.

Taken together, the five-stage sequence shows that the practical value of quantitative SWOT lies not only in identifying or ranking factors, but in strengthening the full transition from contextual clarification and diagnosis to stakeholder-sensitive strategic response.

### 4.1 Practical implications for pharmacy education, industry engagement, and strategic planning

For pharmacy education leaders, the broader implication is that strategic planning should move beyond generic curricular reflection toward more explicit identification of which educational strengths, weaknesses, opportunities, and threats actually carry decision weight. In contexts where programs must respond to changing workforce expectations, digital transformation, and expanding professional roles, descriptive SWOT may help frame the environment, but quantitative SWOT can better support prioritization, stakeholder comparison, and defensible educational planning. Accordingly, the framework may also support strategic decisions in medication-use systems, pharmacy-service implementation, workforce policy, patient-centered practice, and organizational redesign.

This is consistent with pharmacy workforce literature emphasizing the need to align education with evolving professional roles, employability expectations, and technology-enabled practice.^2^, ^4^ In the illustrative application, quantification showed that the program's first-mover identity, differentiated training streams, academic–industry structure, and employability orientation were not interpreted equally by academics, partner managers, and students.

For university–industry engagement, the findings suggest that partnership-based pharmacy training should be assessed not only by whether collaboration exists, but also by how different stakeholders value its benefits, burdens, risks, and implementation requirements. In the empirical application, the structured academic–industry model and industry-informed leadership functioned as shared strategic anchors, whereas partner managers placed greater emphasis on coordination burden, scalability, training depth, standardization, and the perceived business value of the weekly training structure. This indicates that educational relevance alone is not sufficient; partnership models also need to demonstrate feasibility, sustainability, and partner-side value. This interpretation is consistent with stakeholder-sensitive SWOT literature showing that structured scoring can reduce arbitrariness, clarify priorities, and make stakeholder differences more visible.^20^, ^27^ The present framework extends this logic to pharmacy-related strategic planning by encouraging leaders to recognize stakeholder differences before scaling, redesigning, or institutionalizing partnership-based initiatives.

For pharmacy leaders and managers, the main implication is that quantitative SWOT can make strategic planning more actionable by linking prioritization to stakeholder-sensitive response. In the illustrative application, the progression from descriptive SWOT to quantification, stakeholder-gap analysis, and TOWS translation clarified which factors could serve as shared strategic anchors, which were stakeholder-sensitive, and which could be carried forward into SO, WO, ST, and WT strategic options. Shared strengths and opportunities supported SO growth-oriented positioning, whereas coordination burden, single-coordinator dependence, limited standardization, scalability constraints, and partner-side value concerns informed WO reinforcement and WT risk-reduction options. This supports the wider methodological argument that quantitative and TOWS-based SWOT extensions can strengthen decision relevance by moving SWOT from factor listing toward prioritized strategic response.^28^, ^29^ Thus, the framework helps leaders decide what should be protected, strengthened, redesigned, or negotiated with stakeholders, rather than simply producing a longer SWOT list.

To strengthen practical utility, the conceptual framework and empirical example were condensed into **an** 11-step implementation checklist. Table 6 summarizes how pharmacy leaders, managers, and educators can move from contextual mapping and focal SWOT diagnosis to quantification, stakeholder-gap interpretation, factor selection, and TOWS-based strategic response.

**Table 6:** Practical reproducibility checklist for applying quantitative SWOT.

Step	What to do	Practical question	Output	Where illustrated in this paper
**1. Define the focal strategic unit and assign roles**	Specify the organization, program, service, or policy to be analyzed, and assign the analytical lead and decision owner.	What exactly is the strategic unit under study, and who leads the analysis and uses the outputs?	Clear unit of analysis and role assignment	**Concept:** empirical application logic. **Example:** case and background; and Supplementary Tables S1, S2.
**2. Separate macro-context from focal analysis**	Use PESTEL to map the broader environment before building SWOT.	What belongs to the wider environment, and what belongs specifically to the focal case?	Macro-environment profile	**Concept:** SWOT–PESTEL distinction and Table 1. **Example:** SWOT- PESTEL, Table 3 and Supplementary Table 3.
**3. Draft candidate PESTEL and SWOT statements**	Generate initial factors from documents, case material, and implementation evidence.	What strategic conditions and case-specific factors are initially visible?	Candidate factor pool	**Concept:** descriptive SWOT / PESTEL logic. **Example:** methods—documentary sources, data collection, SWOT statement development
**4. Refine and finalize statements for circulation**	Review, de-duplicate, classify, and sharpen statements before stakeholder rating.	Are the statements distinct, non-overlapping, and correctly classified?	Finalized SWOT statements with related codes	**Concept:** internal vs external distinction. **Example:** descriptive SWOT section and Table 3
**5. Define stakeholder groups**	Identify the groups whose judgments matter strategically.	Whose priorities will shape feasibility, implementation, or acceptance?	Stakeholder map	**Concept:** stakeholder-gap extension of quantitative SWOT. **Example:** methods—participants and strategic roles
**6. Quantify baseline importance**	Ask stakeholders to rate each SWOT factor using a common scale.	What does each stakeholder group prioritize?	Mean ratings and ranked factors	**Concept:** quantitative SWOT levels and Table 2. **Example:** baseline ranking section and Supplementary Table 4
**7. Compare stakeholder views**	Test where stakeholder groups converge or diverge.	Which factors are shared, and which are stakeholder-dependent?	Stakeholder-gap profile	**Concept:** baseline quantitative SWOT ratings section as an applied extension. Section 2.4 **Example:**Table 4
**8. Group the quantified profile for interpretation**	Condense the profile into zones of convergence and separation.	Which factors are stable strategic anchors, and which require more cautious interpretation?	Grouped synthesis	**Concept:** gap interpretation logic. **Example:** grouped synthesis and Fig. 3
**9. Select factors for TOWS matrix translation**	Choose a limited subset based on agreement, gap status, and practical actionability.	Which factors are important enough and practical enough to carry into strategy?	Selected factor set	**Concept:** SWOT-to-TOWS transition, Section 2.5 and Fig. 2 **Example:** pre-TOWS selection paragraph, Fig. 3 and Table 5
**10. Translate through TOWS matrix**	Convert selected S/W/O/T factors into SO, WO, ST, and WT options.	How should the organization respond strategically?	TOWS-based strategic options	**Concept:** TOWS section 2.5 and Fig. 2 **Example:** TOWS translation Fig. 3 and Table 5
**11. Use findings for decision support**	Apply outputs to planning, redesign, negotiation, or implementation improvement.	What should be protected, strengthened, corrected, or redesigned first?	Action-oriented strategic interpretation	**Concept:** discussion and practical implications. **Example:** discussion section

This checklist is intended as a practical implementation guide rather than a rigid protocol. Analytic and governance roles are clarified in the methodological table of the empirical application; the present checklist focuses on the applied sequence, outputs, and practical questions involved in moving from descriptive mapping to strategic translation.

### 4.2 Strengths and limitations

A key strength of this article is its conceptual methodological contribution to pharmacy strategy. It extends descriptive SWOT beyond factor listing by integrating prioritization, stakeholder comparison, and TOWS-based strategic translation. It also clarifies the distinction between PESTEL and SWOT, reducing the risk of conflating macro-environmental scanning with focal strategic diagnosis. A further strength is the inclusion of an illustrative pharmacy education application, which demonstrates how the framework can be operationalized in practice rather than presented only as a conceptual roadmap. The application shows the full analytical sequence: contextual mapping, descriptive SWOT, baseline quantification, stakeholder-gap analysis, grouped synthesis, and strategic translation.

Following Lingard's reflective approach, the limitations of this article are best understood as boundaries that define the scope and transferability of the contribution, rather than as defects that negate its value.^57^ The empirical application was designed to demonstrate how the framework can be operationalized, not to provide a definitive evaluation of the focal training model. This means that the case should be read as a methodological illustration of quantitative SWOT in use, rather than as conclusive evidence about the effectiveness or generalizability of the training program itself. The modest and unequal stakeholder groups limit statistical generalization, despite the use of nonparametric testing and adjusted pairwise comparisons. Accordingly, the findings should be interpreted as illustrative stakeholder-prioritization and gap patterns that show how the framework can reveal alignment and separation across groups, rather than as population-level estimates.

The application used baseline importance rating and stakeholder-gap analysis rather than more intensive extensions such as full weighted scoring, AHP, ANP, or fuzzy SWOT.^44^, ^46^, ^49^, ^50^, ^51^ This choice strengthens the feasibility and accessibility of the example, but it also limits the depth of weighting, consistency testing, interdependence modeling, and uncertainty-sensitive prioritization. Similarly, the TOWS matrix was intentionally selective rather than exhaustive; its purpose was to demonstrate how quantified and stakeholder-sensitive findings can be translated into strategic options, not to produce a complete strategy plan for the focal program. Finally, the empirical example was situated in one pharmacy education and labor-market context.

Future studies should apply the proposed conceptual quantitative SWOT framework to more focused strategic questions across pharmacy-related sectors. Such applications could examine how quantitative SWOT reshapes strategic planning in community pharmacy, hospital pharmacy, pharmaceutical industry, regulatory planning, digital pharmacy, and workforce-development initiatives. Future work should move beyond one-time strategic diagnosis by measuring stakeholder-priority gaps systematically and examining the longer-term impact of quantitative, measurable SWOT-based planning on decision-making, implementation alignment, and organizational learning across different organizational, educational, and policy contexts.

## 5 Conclusion

Quantitative SWOT can strengthen pharmacy strategic planning by moving SWOT beyond descriptive classification toward prioritization, stakeholder comparison, and action-oriented translation. The illustrative application demonstrates one way to operationalize the framework, but the central contribution is the transferable methodological pathway.

The take-home message is that quantitative SWOT is valuable not because it adds numbers to a SWOT list, but because it creates a clearer decision pathway: define the focal SWOT profile, separate macro-context from focal strategy, quantify factor importance, identify stakeholder-priority gaps, and translate selected findings into strategic response. For pharmacy leaders, managers, and educators, this provides a scalable framework for making strategic planning more transparent, comparative, and implementation oriented.

## Declaration of generative AI and AI-assisted technologies in the manuscript preparation process

During the preparation of this manuscript, the corresponding author used ChatGPT (OpenAI) as a language-support tool to assist with translation (Arabic–English), editing, clarification of wording, and improvement of coherence and academic writing style. All results and references cited in this manuscript were independently selected, verified, and checked by the authors; no results or references were generated using AI tools.

Following the use of ChatGPT, the authors carefully reviewed, revised, and edited the entire manuscript and take full responsibility for the accuracy, integrity, and originality of the final work.

## CRediT authorship contribution statement

**Mohanad Odeh:** Writing – review & editing, Writing – original draft, Visualization, Supervision, Software, Methodology, Investigation, Formal analysis, Data curation, Conceptualization. **Khaled A. Harb:** Writing – review & editing, Writing – original draft, Validation, Software, Resources, Project administration, Investigation, Formal analysis, Data curation.

## Declaration of competing interest

The authors received no financial support for this research and declare no conflicts of interest.

## References

[1] Morrow N.C. (2015). Pharmaceutical policy Part 1 The challenge to pharmacists to engage in policy development. J Pharm Policy Pract.

[2] Bates I., Patel D., Chan A.H.Y. (2023). A comparative analysis of pharmaceutical workforce development needs across the commonwealth. Res Social Adm Pharm.

[3] Barakat M., Sallam M. (2025). Pharmacy workforce: a systematic review of key drivers of pharmacists’ satisfaction and retention. J Pharm Policy Pract.

[4] Almaghaslah D., Al-Haqan A., Al-jedai A., Alsayari A. (2022). Adopting global tools for the advancement of pharmacy practice and workforce in Saudi Arabia. Saudi Pharm J.

[5] Officer T.N., McDonald J., Smiler K. (2025). Developing integrated extended pharmacist roles and services for equitable access and outcomes in primary healthcare: a realist evaluation. Int J Health Policy Manag.

[6] Martini N., Sajtos L., Idio L. (2024). The future of pharmacy work: how pharmacists are adapting to and preparing for technology infusion. Explor Res Clin Soc Pharm.

[7] Pangarkar N., Prabhudesai R. (2024). Using Porter’s Five Forces analysis to drive strategy. Glob Bus Organ Excell.

[8] Barney J. (1991). Firm resources and sustained competitive advantage. J Manage.

[9] Hammer T. (2022). Value innovation by creating blue oceans. OALib.

[10] Whitehead J. (2015). Wiley Encyclopedia of Management.

[11] Zupic I., Čater T., Caputo A., Uršič D. (2025). Mapping the influence of Ansoff’s corporate strategy. Strateg Chang.

[12] Schutselaars J., Romme A.G.L., Bell J., Bobelyn A.S.A., van Scheijndel R. (2023). Designing and testing a tool that connects the value proposition of deep-tech ventures to SDGs. Designs.

[13] Madsen D.Ø. (2025). Balanced scorecard: history, implementation, and impact. Encyclopedia.

[14] Wowerath C. (2026). Objectives and key results as a tool for middle managers to enhance the process of strategy implementation. J Bus Res.

[15] Schoemaker P.J.S. (1995). Scenario planning: a tool for strategic thinking. Long Range Plann.

[16] Puyt R.W., Lie F.B., Wilderom C.P.M. (2023). The origins of SWOT analysis. Long Range Plann.

[17] Benzaghta M.A., Elwalda A., Mousa M., Erkan I., Rahman M. (2021). SWOT analysis applications: an integrative literature review. J Glob Bus Insights.

[18] Alshahrani S.M. (2026). A mixed-methods SWOT analysis of community pharmacy services in Saudi Arabia: stakeholder perspectives and strategic framework. Front Med.

[19] Krylova O., Litvinova T., Babaskina L., Babaskin D., Savinova O. (2020). Analysis of the internal environment of the pharmaceutical distributor operation in Russia using SWOT analysis. Open Access Maced J Med Sci.

[20] Stacchini A., Guizzardi A., Mariotti A. (2022). Smoothing down arbitrariness in planning: from SWOT to participatory decision making. Land Use Policy.

[21] Battulga S., Dhakal S. (2024). Stakeholders’ perceptions of sustainable energy transition of Ulaanbaatar city. Mongolia Renew Sustain Energy Rev.

[22] Helms M.M., Nixon J. (2010). Exploring SWOT analysis – where are we now?. J Strateg Manag.

[23] Mircescu G.D. (2023). Analysis of the external environment of the pharmaceutical organization. Logos Univ Ment Educ Nov Soc Sci.

[24] Halmosi P., Aranyossy M. (2024). Exploring macro-environmental catalysts and barriers of healthcare 4.0 transformation in Central-Eastern European countries: a comprehensive study in Hungary. Technol Soc.

[25] Vardopoulos I., Tsilika E., Sarantakou E., Zorpas A., Salvati L., Tsartas P. (2021). An integrated SWOT-PESTLE-AHP model assessing sustainability in adaptive reuse projects. Appl Sci.

[26] Moroz S., Sahaidak-Nikitiuk R., Zoidze D. (2019). Implementation of the quantitative SWOT analysis method in the research on the contemporary state and prospects of consistent development of the pharmaceutical branch. Res J Pharm Technol.

[27] Phadermrod B., Crowder R.M., Wills G.B. (2019). Importance-performance analysis based SWOT analysis. Int J Inf Manag.

[28] David M.E., David F.R., David F.R. (2017). The quantitative strategic planning matrix: a new marketing tool. J Strateg Mark.

[29] Weihrich H. (1982). The TOWS matrix—a tool for situational analysis. Long Range Plann.

[30] Đalić I., Stević Ž., Ateljević J., Turskis Z., Zavadskas E.K., Mardani A. (2021). A novel integrated MCDM-SWOT-TOWS model for the strategic decision analysis in transportation company. Facta Univ Ser Mech Eng.

[31] Crul M., Polidori C., Paolucci D. (2024). Centralization and automation of non-toxic drug reconstitution in the pharmacy: a strengths, weaknesses, opportunities, and threats analysis. Int J Pharm Pract.

[32] de Castro Araújo-Neto F., Dosea A.S., da Fonseca F.L. (2024). Formal leadership perceptions about the autonomy of pharmacy: a SWOT analysis. Explor Res Clin Soc Pharm.

[33] Cahyana A.A., Harsono S.B., Herdwiani W. (2025). SWOT analysis with ServQual model of pharmaceutical services at RSUD Ibu Fatmawati Soekarno Surakarta in 2024. Formosa J Multidiscip Res.

[34] Suwanti A., Yasin N.M., Rahayu W.S. (2025). Development strategy of the pharmacy department at Nirmala Hospital based on SWOT analysis. J Heal Econ Policy Res.

[35] Almeman A.A. (2020). Strategic analysis of clinical pharmacy education in Saudi Arabia. Trop J Pharm Res.

[36] Pires C. (2023). A SWOT analysis of pharmacy students’ perspectives on e-learning based on a narrative review. Pharmacy.

[37] Rhoney D.H., Chen A.M.H., Thornby K.A. (2026). Assessing the current state of pharmacy education: a strengths-weaknesses-opportunities-threats analysis. Am J Pharm Educ.

[38] Murthy M.B., Ramanand S., Karande V.B., Gaidhankar S., Sakhare P. (2025). SWOT analysis for strategy building to improve learning outcomes of prescription communication in medical students. Southeast Asian J Med Educ.

[39] Ulianova I.E., Egorova S.N. (2025). Analysis of the potential and prospects for selling pharmacy products via the internet. Bull Contemp Clin Med.

[40] Wahab S.N., Ahmed N., Ab Talib M.S. (2024). An overview of the SWOT analysis in India’s pharmaceutical supply chain. Arab Gulf J Sci Res.

[41] Zaurez Afshar M., Hussain Shah D.M. (2025). Strategic evaluation using PESTLE and SWOT frameworks: public sector perspective. SSRN Electron J.

[42] Morita P.P., Abhari S., Kaur J., Lotto M., Padses Miranda, Oetomo A. (2023). Applying ChatGPT in public health: a SWOT and PESTLE analysis. Front Public Health.

[43] Solanki R., Kennedy C., Shirke Y. (2022). 2022 ASEE Annual Conference & Exposition Proceedings.

[44] Abdel-Basset M., Mohamed M., Smarandache F. (2018). An extension of neutrosophic AHP–SWOT analysis for strategic planning and decision-making. Symmetry (Basel).

[45] Wang M., Yu W. (2023). Proceedings of the 4th International Conference on Economic Management and Model Engineering, ICEMME 2022, November 18-20, 2022, Nanjing, China.

[46] Kurttila M., Pesonen M., Kangas J., Kajanus M. (2000). Utilizing the analytic hierarchy process (AHP) in SWOT analysis — a hybrid method and its application to a forest-certification case. Forest Policy Econ.

[47] Lee H., Kim C., Cho H., Park Y. (2009). An ANP-based technology network for identification of core technologies: a case of telecommunication technologies. Expert Syst Appl.

[48] Shakoor Shahabi R., Basiri M.H., Rashidi Kahag M., Ahangar Zonouzi S. (2014). An ANP–SWOT approach for interdependency analysis and prioritizing the Iran’s steel scrap industry strategies. Resour Policy.

[49] Liou T.S., Wang M.J.J. (1992). Ranking fuzzy numbers with integral value. Fuzzy Set Syst.

[50] Chang D.Y. (1996). Applications of the extent analysis method on fuzzy AHP. Eur J Oper Res.

[51] Liu Y., Eckert C.M., Earl C. (2020). A review of fuzzy AHP methods for decision-making with subjective judgements. Expert Syst Appl.

[52] Stević Ž., Stjepanović Ž., Božičković Z., Das D.K., Stanujkić D. (2018). Assessment of conditions for implementing information technology in a warehouse system: a novel fuzzy PIPRECIA method. Symmetry (Basel).

[53] Ghazinoory S., Abdi M., Azadegan-Mehr M. (2011). SWOT methodology: a state-of-the-art review for the past, a framework for the future / SSGG metodologija: praeities ir ateities analizė. J Bus Econ Manag.

[54] Rui S., Makanae K., Fujiu M., Morisaki Y. (2024). SWOT-AHP analysis of BIM technology utilization in the Japanese construction industry. Buildings.

[55] Skotnicka-Zasadzien’ B., Zasadzien’ M., Grebski W. (2023). Application of TOWS/SWOT analysis as an element of strategic management on the example of a manufacturing company. Sci Pap Silesian Univ Technol Organ Manag Ser.

[56] Nguyen T., Truong C. (2022). Integral SWOT-AHP-TOWS model for strategic agricultural development in the context of drought: a case study in Ninh Thuan, Vietnam. Int J Anal Hierarchy Process.

[57] Lingard L. (2015). The art of limitations. Perspect Med Educ.

