# Supplementary material for: Beyond descriptive SWOT in pharmacy strategy: A quantitative framework for leaders, managers and educators from prioritization to strategic response
Source: Explor Res Clin Soc Pharm. 2026 Aug 24;24:100836. doi: 10.1016/j.rcsop.2026.100836 (PMC13573747; doi:10.1016/j.rcsop.2026.100836)
Supplement: Supplementary file 1 [file mmc1.docx]

Supplementary Table S1. Operational profile of the illustrative empirical application: a structured university–industry advanced training model for final-year pharmacy students

## Short contextual background

Jordanian pharmacy education is offered through 17 schools of pharmacy, including 5 public and 12 private institutions. To qualify for pharmacist practice, students are generally required to complete extensive field training, typically around 1440 hours under local professional expectations. In most schools, experiential training is centered largely in community pharmacy, with more limited hospital exposure, whereas PharmD programs follow a distinctly clinical orientation that includes a full hospital-based training year. Although only a few schools (3 schools) have offered PharmD training, the standard pharmacy degree remains the dominant national model. Within this context, three newer public schools established between 2011 and 2013 introduced a track-based approach intended to provide additional specialization, particularly in Administrative (Management) Pharmacy and Industrial Pharmacy. Over time, however, only one school retained this model. These tracks do not alter graduates’ status as fully qualified pharmacists able to work across sectors; rather, they provide a 12-credit-hour concentration over last semester to build enhanced competencies in a specific domain. The present empirical case focuses on an advanced training model linked specifically to the Administrative (Management) Pharmacy track.

Pharmacy education and practice in Jordan are already well described in the literature; therefore, only the contextual features most relevant to the present case are outlined here. This empirical application is presented as a practical example of advanced quantitative SWOT in pharmacy education, with broader relevance beyond Jordan.

## Case (what, who, how, when, where, why, duration, structure, and participants selection)

The empirical application focused on a unique structured university–industry advanced training model for final-year pharmacy students in the Administrative Pharmacy track at the Hashemite University. In its concept form, the program targeted approximately 30–35 students and was scheduled over 6–12 weeks, typically across one academic semester at 2 days per week and 8 hours per day. Candidate selection was based on university nomination followed by joint academic–industry interviewing. In the applied model, training was delivered through multiple partner institutions rather than a single host site, with student allocation determined by academic screening, interview outcomes, program requirements, and expected employability fit.

Before placement, the program lead delivered a training-of-trainers component for partner supervisors to align supervisory expectations with the knowledge base and learning framework already introduced to students. Each student’s training map was then co-designed by the academic lead and partner supervisor to ensure structured exposure, task relevance, and close alignment between academic preparation and field practice. Delivery occurred across multiple sites, including partner facilities and related practice environments. The program comprised three principal streams: supportive logistics services; planning, development, training, and marketing research; and promotion, communication, awareness, and sales, the latter emerging as the most prominent employability-oriented component in the applied model.

| **Supplementary Table S2. Methodological components of the illustrative empirical application and their operationalization in the focal case** | | |
| --- | --- | --- |
| **Method component** | **General methodological function** | **Applied example in the focal case** |
| **Design** | Define the overall empirical approach | Mixed-methods, cross-sectional, stakeholder-based application of advanced quantitative SWOT |
| **Setting** | Define the institutional and operational context of the empirical application | University–industry advanced training model in Administrative Pharmacy at the Hashemite University, implemented across multiple partner sites and related practice environments |
| **Participants**  **and strategic roles** | Identify the stakeholder participant groups and clarify analytic and decision roles | The empirical application involved three stakeholder participant groups: trainees (representative students), the academic side, and partner managers/industry-side representatives. The analytical lead was responsible for structuring the process, drafting and refining the candidate PESTEL and SWOT statements, organizing the quantitative analysis, and interpreting the resulting profile. The decision owner role referred to the program side responsible for using the outputs to guide strategic interpretation and future implementation direction. |
| **Sample size and stakeholder distribution** | Clarify the final analytic sample and its distribution across stakeholder groups | The final analytic sample included 29 valid respondents across three stakeholder groups: academics (n = 5), managers/partner representatives (n = 16), and students/trainees (n = 8). |
| **Documentary sources** | Establish the formal structure and implementation logic of the case | Program annex, planning materials, and implementation documents |
| **Data collection and analysis** | Specify how strategic factors were generated and how judgments were collected | Separate stakeholder-group sessions were conducted with academics, students, and field supervisors. Using program documents and real implementation examples, structured brainstorming was used to generate the initial PESTEL and SWOT factors. Final SWOT statements were then distributed through a Google Form for stakeholder rating. |
| **Macro-context layer** | Position the case within its wider external environment | PESTEL summary of Jordanian pharmacy education, labor market, and pharmacy-related professional system |
| **SWOT statement development** | Convert raw evidence into final strategic statements | Candidate statements were generated from documents, real case examples, and stakeholder brainstorming, then refined, de-duplicated, and classified into strengths, weaknesses, opportunities, and threats |
| **Quantification** | Translate finalized SWOT statements into comparable priorities | Baseline ordinal rating of finalized SWOT factors using a common 5-point importance scale. Ratings were used to compare stakeholder priorities and identify stakeholder-gap patterns. No formal weighting, magnitude × importance composite score, AHP, ANP, or fuzzy weighting was applied in this illustrative example. |
| **Statistical analysis** | Specify how stakeholder ratings were summarized and compared analytically | SWOT ratings were summarized as mean (standard deviation) for comparative ranking across stakeholder groups. Inter-group differences were examined using the Kruskal–Wallis test, with Bonferroni-adjusted pairwise comparisons used to interpret the direction and strength of stakeholder gaps. |
| **Stakeholder comparison** | Identify agreement and divergence across groups | Ratings were compared across trainees, academic participants, and partner supervisors to identify shared priorities and stakeholder gaps |
| **Main output** | Clarify what the method produces | PESTEL context, descriptive SWOT, quantification, stakeholder-gap profile, and illustrative TOWS-based strategic options |
| **Strategic translation** | Convert quantified SWOT priorities into actionable strategic directions | Based on the quantified SWOT results, a stakeholder divergence profile was developed, and the analytical lead translated the selected findings into TOWS-based strategic options intended to inform the decision owner |
| **Ethical considerations** | Clarify ethical review, approval, and oversight of the empirical application | The empirical application part for the student training and evaluation was reviewed and approved by the Institutional Review Board / Ethics Committee of the Hashemite University (approval number: 2/2/2019/2020). |

Supplementary Table S3. PESTEL analysis of the Jordanian pharmacy education environment, labor market, and pharmacy-related professional system

P — Political / Policy

-Centralized governance shapes pharmacy education, recognition, and admissions in Jordan.

-National policy increasingly favors employability and practice-linked education.

-Pharmacy-program innovation is shaped by modernization and professional-readiness expectations.

-Jordan’s relative political and security stability supports educational continuity and partnership building.

E — Economic

-The pharmaceutical sector is economically important and export-active.

-National unemployment remains high, increasing pressure for job-ready graduates.

-Pharmacy careers extend beyond community practice to industry, marketing, regulation, quality, and hospitals.

- Some non-traditional pharmacy roles may offer comparatively attractive career opportunities.

-In the Jordanian labor market, medical representative roles are perceived as among the most financially attractive early-career options, not only within pharmacy but also relative to many other graduate-entry jobs during the first years after graduation.

S — Social

-Employers increasingly expect communication, teamwork, leadership, and adaptability alongside scientific knowledge.

-A gap persists between traditional academic preparation and applied interpersonal role demands.

-Pharmacy graduates in Jordan enter diverse career pathways rather than a single track.

-Pharmacy education appears to have a predominantly female student profile, which may influence career expectations and workforce patterns.

-Track-based education remains limited in Jordanian pharmacy schools, despite its potential value for earlier career orientation and occupational identity formation.

T — Technological

-Jordan’s pharmaceutical governance includes electronic registration and online submission systems.

-Pharmacy practice and pharmacy-related work in Jordan increasingly rely on digital systems, electronic workflows, and technology-supported tasks.

-Well-developed pharmacy management software is increasingly available for dispensing, inventory, reporting, and administrative functions.

-In industry- and promotion-facing roles, CRM, tracking, and customer-management systems support marketing coordination, field follow-up, and performance monitoring.

-Webinars and online educational activities for healthcare providers have expanded digital channels for pharmaceutical communication, professional education, and promotional engagement.

E — Environmental/Sectoral Environment

-Pharmacy in Jordan operates across community, hospital, industry, warehouse, and regulatory settings.

- In addition to traditional employer for pharmacists, employment spans multiple actors, including local manufacturers, international companies, company agents, wholesalers, subagents, and smaller sectors such as insurance, freelance training/education, and research centers.

-Sector needs extend beyond dispensing to supply, regulation, quality, communication, and organization.

-This diversity increases the value of function-specific, real-world training.

L — Legal / Regulatory

-Pharmacy practice and pharmaceutical-sector activity in Jordan are governed by formal regulatory and professional frameworks involving the Ministry of Health, JFDA, and pharmacist registration structures.

-In the Jordanian context, health promotion and pharmaceutical marketing roles are primarily pharmacist-based, with non-pharmacists not typically authorized to work as medical representatives.

-Prescription medicines are subject to stricter pricing and promotional control, including restriction of promotion to specialized healthcare providers rather than the general public.

-Supplements, medical devices, and cosmetics operate under different promotional conditions, with relatively broader scope for direct-to-patient communication than prescription medicines.

-Pharmacy graduates therefore enter a profession in which compliance, product classification, licensing, and promotional boundaries are central to practice.

-Qualification for pharmacist practice in Jordan is linked to mandatory field training requirements, typically involving **1440 hours** of supervised training documented within university and professional structures, including JPA-related oversight.

| **Supplementary Table S4. Baseline quantitative SWOT ratings: what did each stakeholder group prioritize?** | | | | | |
| --- | --- | --- | --- | --- | --- |
| **Code** | **SWOT factor** | **Academics (n=5) mean (SD)** | **Managers (n=16)**  **mean (SD)** | **Students (n=8)**  **mean (SD)** | **Kruskal–Wallis P** |
| S1 | The program provides structured field-based training beyond routine placement. | 5.00  (0.00) | 4.00 (1.10) | 4.88 (0.35) | .021 |
| S2 | The program represents a first-mover and uniquely tailored Administrative Pharmacy training model within the Jordanian pharmacy education context. | 5.00  (0.00) | 4.00 (1.03) | 4.88 (0.35) | .011 |
| S3 | The program includes three differentiated training streams. | 5.00  (0.00) | 3.19 (1.47) | 4.62 (0.52) | .001 |
| S4 | The program uses a joint academic–industry partnership model with Training of Trainers induction, tailored preparation, and student-specific training maps. | 5.00  (0.00) | 4.56 (0.63) | 5.00 (0.00) | .053 |
| S5 | The program benefits from leadership with direct industry experience. | 5.00  (0.00) | 4.88 (0.34) | 5.00 (0.00) | .431 |
| S6 | The curriculum provides two protected training days per week. | 5.00  (0.00) | 2.44 (1.63) | 4.75 (0.46) | <.001 |
| W1 | The program has a high coordination burden across partners and sites. | 4.60  (0.55) | 4.12 (1.09) | 2.38 (0.74) | .001 |
| W2 | The program depends heavily on one academic coordinator for follow-up. | 4.80  (0.45) | 4.12 (1.26) | 2.13 (0.83) | .001 |
| W3 | Implementation and monitoring are not fully standardized across sites. | 4.40  (1.34) | 4.12 (1.20) | 2.50 (1.31) | .017 |
| W4 | The two-day field format may limit training depth. | 2.20  (1.64) | 3.81 (1.64) | 2.12 (1.13) | .039 |
| W5 | The program has limited dedicated funding for partner and student training costs. | 5.00  (0.00) | 4.38 (1.15) | 4.12 (1.36) | .325 |
| W6 | The program may be difficult to scale because of transport, location, and mobility constraints. | 2.40  (1.67) | 4.50 (0.82) | 3.38 (1.60) | .020 |
| O1 | The program can benefit from growing demand for non-traditional pharmacy careers. | 5.00  (0.00) | 4.25 (0.77) | 4.75 (0.71) | .040 |
| O2 | The program can expand through additional strategic partners. | 5.00  (0.00) | 4.19 (0.66) | 4.75 (0.46) | .012 |
| O3 | The program can engage companies that benefit from student trainees. | 5.00  (0.00) | 3.13 (1.41) | 4.62 (0.74) | .002 |
| O4 | The program can use digital tools, CRM systems, and webinars to strengthen training. | 5.00  (0.00) | 2.75 (1.73) | 4.75 (0.46) | .003 |
| O5 | The program can improve university-to-work transition pathways. | 5.00  (0.00) | 4.50 (0.52) | 4.62 (0.52) | .142 |
| O6 | The program can strengthen the visibility of Administrative Pharmacy. | 5.00  (0.00) | 4.44 (0.51) | 4.62 (0.52) | .088 |
| T1 | Other universities may replicate the model and compete for partner slots. | 4.60  (0.89) | 2.75 (1.24) | 4.38 (0.74) | .003 |
| T2 | Labor-market pressure may increase expectations for visible employability outcomes. | 4.80  (0.45) | 3.75 (1.13) | 4.38 (0.74) | .092 |
| T3 | Conventional community- and hospital-based training expectations may limit support for this model. | 4.80  (0.45) | 3.88 (1.09) | 3.62 (1.30) | .162 |
| T4 | Variation across partner practices may create unequal training exposure. | 2.80  (2.05) | 4.25 (0.93) | 3.12 (1.36) | .106 |
| T5 | Because training is not the partners’ primary mission, student activities may be seen as a burden. | 3.40  (2.19) | 3.94 (1.24) | 3.12 (1.36) | .412 |
| T6 | The limited weekly time structure may reduce perceived business value for partners. | 2.80  (2.05) | 4.06 (1.29) | 3.00 (1.41) | .159 |
| Ratings were collected on a 5-point ordinal importance scale, with higher values indicating greater perceived importance. Values are presented as mean (SD) only as descriptive prioritization scores to support comparison across multiple SWOT factors and stakeholder groups. P values were derived from Kruskal–Wallis tests comparing academics, partner managers or industry representatives, and students or trainees. Findings are interpreted as illustrative stakeholder-prioritization patterns rather than generalizable estimates because of the small, unequal stakeholder groups. SWOT = Strengths, Weaknesses, Opportunities, and Threats; S = strength; W = weakness; O = opportunity; T = threat. | | | | | |
